# Mechanical Properties of Protein-Based Food Packaging Materials

**DOI:** 10.3390/polym15071724

**Published:** 2023-03-30

**Authors:** Yasir Abbas Shah, Saurabh Bhatia, Ahmed Al-Harrasi, Muhammad Afzaal, Farhan Saeed, Md Khalid Anwer, Mahbubur Rahman Khan, Muhammad Jawad, Noor Akram, Zargham Faisal

**Affiliations:** 1 Natural & Medical Sciences Research Center, University of Nizwa, P.O. Box 33, Nizwa 616, Oman; yasir.shah@unizwa.edu.om (Y.A.S.); m.jawad@unizwa.edu.om (M.J.); 2 School of Health Science, University of Petroleum and Energy Studies, Dehradun 248007, India; 3 Centre for Transdisciplinary Research, Department of Pharmacology, Saveetha Dental College, Saveetha Institute of Medical and Technical Science, Chennai 600077, India; 4 Department of Food Science, Government College University, Faisalabad 38000, Pakistan; muhammadafzaal@gcuf.edu.pk (M.A.); f.saeed@gcuf.edu.pk (F.S.); noorakram462@gmail.com (N.A.); 5 Department of Pharmaceutics, College of Pharmacy, Prince Sattam Bin Abdulaziz University, Al-kharj 11942, Saudi Arabia; m.anwer@psau.edu.sa; 6 Department of Food Processing and Preservation, Hajee Mohammad Danesh Science & Technology University, Dinajpur 5200, Bangladesh; emon.1707047@std.hstu.ac.bd; 7 Institute of Food Science and Nutrition, Bahauddin Zakariya University, Multan 60000, Pakistan; zarghamfaisal14@gmail.com

**Keywords:** food packaging, protein, mechanical properties, edible films, biopolymers

## Abstract

The quality and safety of food products greatly depend on the physiochemical properties of the food packaging material. There is an increasing trend in the utilization of protein-based biopolymers for the preparation of edible films and coating due to their film-forming properties. Various studies have reported the preparation of protein-based edible films with desirable mechanical and barrier properties. The mechanical attributes of the protein-based food packaging materials can be enhanced by incorporating various components in the film composition such as plasticizers, surfactants, crosslinkers, and various bioactive compounds, including antimicrobial and antioxidant compounds. This review article summarizes the recent updates and perspective on the mechanical attributes such as Tensile Strength (TS), Elongation at Break (EAB), and Young’s Modulus (YM) of edible films based on different proteins from plants and animal sources. Moreover, the effects of composite materials such as other biopolymers, bioactive compounds, essential oils, and plasticizers on the mechanical properties of protein-based edible films are also discussed.

## 1. Introduction

There is a great demand for the development and utilization of edible films based on natural polymers due to the harmful effects of plastic-based packaging materials. Edible films have great potential to be utilized as food packaging for the freshness, safety, shipping, storage, and presentation of various foods. Edible films made from natural polymers such as carbohydrates, lipids, and proteins are safe for human consumption and do not pose any health risks. Because of these advantages, edible packaging has become more popular as a replacement for both synthetic and natural degradable plastic food packaging [1].

Edible films must possess excellent mechanical and barrier properties to replace plastic based packaging material at industrial level. The mechanical and barrier characteristics of edible films are of the utmost importance because of the significant impact they have, either directly or indirectly, on the food product. Tensile Strength (TS), elongation at Break (EAB), and Young’s Modulus (YM) are the three most crucial parameters to consider when examining the edible films for their mechanical properties [2]. The mechanical attributes of edible films depend on different factors such as the nature of the polymer, preparation method, and the composition of film. The mechanical properties also affect other parameters of films, such as crystallinity, barrier properties, and thermal stability. 

Proteins are preferred over lipids and carbohydrates for the preparation of edible films to achieve desired film properties. Protein secondary, tertiary, and quaternary structures can be easily modified to achieve excellent film characteristics using various treatments, including thermal denaturation, chemical hydrolysis, enzymatic treatment, and chemical crosslinking [3]. Different types of protein polymers derived from plants (soy protein, zein corn, wheat gluten) and animals (gelatin, casein, whey protein) have been used to form edible films. Studies have demonstrated that protein-based edible films are the most attractive and provide mechanical stability compared to carbohydrates and lipids [4]. Proteins have a specific structure that possesses various functional properties, particularly high intermolecular binding potential, and thus have stronger mechanical properties than carbohydrates and lipids [5].

Blending protein-based polymers with other biopolymers is a promising approach to enhance the properties and functionality of edible films. The physicochemical characteristics of fish-gelatin-based films with chitosan addition were investigated by Hosseini et al. [6]. The findings revealed a considerable enhancement in the tensile strength (TS) and elastic modulus of the resulting films, thereby imparting superior mechanical properties compared to pure gelatin films. Similarly, incorporation of essential oils and plant extracts can also exert an influence on the mechanical properties of protein-based polymers. In comparison to composite films containing lipids, simple protein films exhibit greater strength and extensibility [7]. The impact of Nigella sativa essential oil (NSEO) on the physicochemical characteristics of milk-protein-based edible films was examined by Ghamari et al. [8]. The mechanical testing revealed that the incorporation of NSEO resulted in a decline in the tensile strength and Young’s modulus of the films, whereas the elongation at break increased in proportion to the amount of NSEO added. Several studies have also reported a decrease in the tensile strength of the edible films with the addition of essential oils [2,9,10]. Moreover, the addition of plant extracts and their effect on the mechanical properties of edible films have been reported by many studies. Adilah et al. [11] reported an increase in the tensile strength of soy protein isolate and fish gelatin films when incorporated with mango kernel extracts. The effectiveness and physicochemical characteristics of protein-based films greatly depend on the unique properties of the added constituents as well as film-forming technique [12]. This review provides information on the recent progress in the mechanical properties of different protein-based film-forming materials.

## 2. Mechanical Attributes of Edible Films

The evaluation of mechanical attributes is essential for food packaging because it enables the possible loss-free distribution of food products during transportation and storage while providing a sufficient level of protection [13]. The behavior of the material against stress applied to its surface is correlated with the mechanical attributes of packaging films. Packaging materials must be mechanically strong and extensible enough to withstand manufacturing and consumer stresses. The tensile strength (TS), Young’s modulus (EM), elongation at break (EAB), and activation energy (Ea.) are some of the distinct mechanical properties that are necessary for the edible packaging films to be feasible. These parameters are evaluated to adequately assess the mechanical parameters of an edible packaging film [14].

Tensile strength (TS) and elongation at break (EAB) are the two critical parameters commonly used to evaluate the mechanical properties of prepared edible films. These two factors are strongly correlated with the chemical makeup of the packaging material. Tensile strength is a mechanical property of edible films that represents the maximum stress the film can endure before breaking. Tensile strength is typically denoted by σ and expressed in units of MPa per mole. Elongation at break (%) is the capacity of the film to withstand shape changes without breaking. For edible films, a high elongation is preferred since it enhances the film’s capability to envelop and wrap food [15]. Young’s modulus provides information about the stiffness of the material and explains how the flexibility and mechanical characteristics of a film correlate to its chemical composition. Young’s modulus is denoted by E or Y and expressed in units of GPa [4].

**Figure 1:**
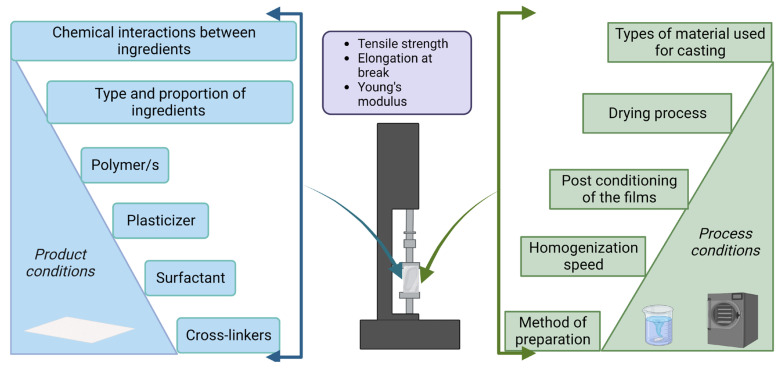
Different factors affecting the mechanical properties of the edible films.

The pH value has been observed to exhibit a favorable effect on the elongation and tensile strength of food packaging film. Edible films produced under alkaline conditions appeared to be more reliable because of hydrogen bonds, disulfide bonds, and hydrophobic bonds, demonstrating that covalent bonding contributes to the endurance of films. Higher pH levels may enhance the mechanical properties of packaging films [16]. A strong oxygen barrier inhibits moisture loss between food and the environment, which enhances the tensile strength and flexibility of edible films. Factors including the solvent being used to dissolve the polymer for the film-forming solution and the molecular weight of the components significantly affect the oxygen barrier properties of food packaging films [17,18]. The variation in the mechanical attributes of edible films can be observed due to different factors (Figure 1) such as the polymer’s origin, manufacturing process, thickness, and exposure to elements that induce changes in the polymer’s structure, including moisture, heat, light, and others. Furthermore, incorporating bioactive and antibacterial compounds in food packaging materials can alter the mechanical attributes [13].

## 3. Assessment of the Mechanical Properties

Mechanical strength of the food packaging material is an essential parameter to determine the strength of the material in an order to check its capability in bearing the resistance or stress offered by the environmental factors and packed food. Several factors such as molecular weight, type, and amount of the polymer play an important role in determining the mechanical strength of the edible films. Similarly, type and proportion of surfactant, plasticizer, and bioactive substance also impact mechanical strength of the edible films. Crystallinity and tensile strength of the packaging material are directly correlated with each other. To assess the mechanical strength of the polymeric materials including edible films for food applications, several tests such as sliding friction, blend and flexure, viscous polymer measurement, tearing strength, shear, fatigue, fracture, indentation, compression, puncture, and tension measurements could be performed. These parameters can be used to assess the mechanical properties such as tensile strength, toughness, elasticity, brittleness, stiffness, softness, adhesiveness, hardness, gel strength, bloom strength, rupture force, burst strength, peeling properties, etc. For mechanical strength assessment, it is important to explore the tensile testing method to understand the behavior of the polymeric material under stress. Generally, tensile testing of packaging material is executed when a load in the form of tension is applied on the polymeric material (also called stress) and it shows deformation, which means the change in the length of the material under stress during testing. This deformation is expressed as percentage strain. Tensile testing is usually performed in two ways, either under fixed load or constant deformation. Tensile strength, loading force, elasticity, puncture strength, and elongation at break play an important role in the mechanical properties of food packaging materials. Tensile strength is considered an important mechanical parameter of packaging materials, as good tensile strength is important for polymeric materials and their mobility. Elongation at break is the extent of a film’s ductility. This parameter reveals how much a film can be stretched. Minimal ductileness of films suggests that it is brittle and thus can be fractured effortlessly under a tensile load. Texture analyzer helps in assessing hardness, extensibility, brittleness, spread ability, adhesiveness, tensile strength, etc. There are several texture analyzers available and used in recent works. Recent studies showed the utilization of different texture analyzers to assess the mechanical strength of the food packaging material as mentioned below in Table 1.

## 4. The Effect of Crystallinity on Mechanical Properties

Mechanical properties are greatly influenced by the extent of the crystallinity of the polymer. Polymers with a high crystalline property present more mechanical and thermal stability. However, low molecular weight polymers usually present weaker strength because of their high crystalline nature [28]. Polymer can be highly crystalline, semicrystalline, or amorphous with variable tendency to crystallize [29,30].

Properties such as crystal size and percentage of crystallinity and amorphousness can be determined by x-ray diffraction analysis (XRD). Characteristic peaks in the XRD graph usually present the high crystallinity at that specific region [31]. The mechanical, crystalline, and deformation behavior (elastic region, both elastic–plastic region, and the plastic region) of polymer is interrelated with each other. These factors are mainly dependent on the microscopic structure and the crystalline morphology of the polymer [32]. The crystalline polymers are relatively brittle compared to amorphous polymers.

Polymers having crystallinity of more than 80% are considered crystalline, however, polymers with crystallinity less than 40% are amorphous. The degree of crystallinity of any polymer is dependent on various factors such as chain length, extent of branching, intermolecular forces, rate of nucleation, and tacticity or stereoisomerism. Usually, long chain polymers present a high degree of polymerization; however, they are less likely to crystallize. Such long chain polymers are more likely to become intertwined and form amorphous regions. Thus, comparatively short polymer chains form crystals more easily than long chain polymers [33]. Similarly, branched chain polymer are less likely to crystallize. On the other side, copolymers such as branched, block, graft, and random show less crystallinity, whereas alternate shows high crystallinity. In the alternate polymer, monomers are arranged alternatively and have inherent periodicity which show higher crystallinity than others, as this structure allows more folding and arrangement. Tacticity or stereoisomerism is an important factor that impacts the crystallinity of the polymer [34].

Atactic polymers have random side groups which do not allow folding, and thus show less crystallinity, whereas syndiotactic and isotactic polymers show the crystallinity. Low molecular weight additives such as plasticizer and especially glycerol prevent crystallization by keeping chains separated from each other. Thus, the addition of plasticizer always improves amorphousness and reduces crystallinity. The number of amorphous or crystalline regions, extend of chain folding, and crystal size also impact the crystallinity of the polymer.

**Figure 2:**
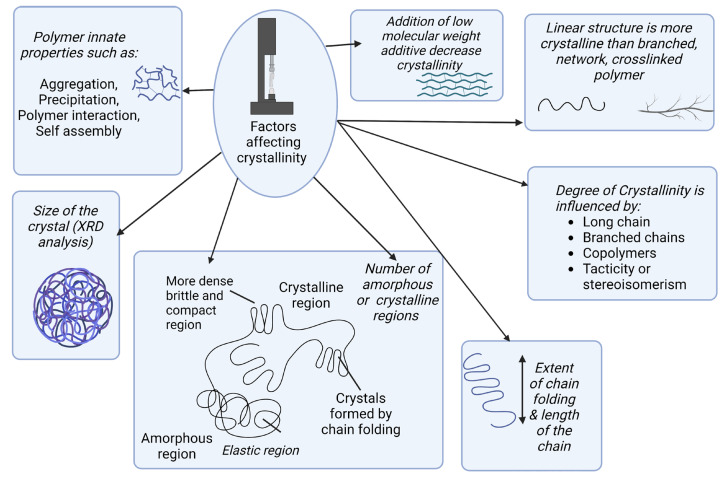
The effect of crystallinity on the mechanical attributes of edible films.

Several factors impact the mechanical strength of the composite polymer (Figure 2). These factors are categorized into two groups. product and process-related parameters significantly impact the mechanical strength of the polymer. Factors such as product composition, proportion of each ingredient, and interaction between these ingredients considerably impact the polymer properties. Product-related parameters such as type and concentration of polymer, surfactant, plasticizer, and cross-linkers impact the mechanical strength of the polymer. Process conditions such as duration and extent of drying and homogenization speed, post conditioning of the films, type of material used for casting, etc., considerably influence edible film properties.

## 5. Mechanical Properties of Plant-Based Protein

Plant-based proteins including soy protein, gliadin, and zein have been utilized to develop edible films with desirable properties. Different characteristics of the proteins, such as conformation, surface hydrophobicity, and thermal stability all play essential roles in film formation [35,36]. Protein films can also be incorporated with a range of bioactive substances since they contain hydrophilic and hydrophobic areas [37]. Because of the forming of a hydrogen bond network structure between polar groups, the active edible film possesses good mechanical and barrier properties. The film-forming potential and features of these substances can be linked to their high mechanical qualities, cross-linking activities, and biodegradability of these substances [38]. Table 2 presents the effect of different composite materials on the mechanical properties of edible films based on plant proteins.

### 5.1. Soy Protein

Soy protein is derived from soybeans and has a range of health benefits. It is regarded as a complete protein among the various dietary proteins because it contains enough quantities of all of the essential amino acids as well as a wide variety of other nutrients [39]. Soy protein is an easily accessible, affordable, biodegradable, and nutrient-rich raw material for preparing edible films. Studies have demonstrated that films based on soy protein are fairly brittle, have a slight beany flavor, and have poor mechanical and physical properties [40]. Cao et al. [40] examined the mechanical attributes of soy protein isolate (SPI) film and reported that the film had poor mechanical properties and was brittle and hard to handle. When SPI and gelatin composite films were prepared, the TS and EAB significantly improved [40]. Nandane et al. [4] investigated the mechanical parameters of the SPI films in relation to the concentration of the SPI. The mechanical parameters, including TS, EAB, and YM of the films, increased with increasing the concentration of SPI. At an SPI concentration of 10 and 6%, the maximum and minimum tensile strengths were found to be 3.5 MPa and 1.6 MPa, respectively. Moreover, due to the intrinsic hydrophilicity of proteins, soy protein films offer only a moderate level of resistance to the transport of water vapors [41].

The preparation of composite films is another method for enhancing the mechanical attributes of the soy protein. Combining soy protein films with starch, sodium alginate, and whey protein isolate can improve the physiochemical properties of the films [42,43].

### 5.2. Zein Corn

Zein is a hydrophobic prolamin protein found in corn. Zein has been utilized in the preparation of edible films due to its excellent film-forming properties [44]. Corn-zein proteins could be appropriate as a barrier protective film for food packing because of its excellent barrier properties [45]. It has been reported that films based on zein show brittle nature in terms of mechanical properties [46]. Due to their brittleness, different plasticizers are utilized in the fabrication of zein films to provide flexibility. Torun et al. [47] studied the mechanical parameters of zein- and milk-protein-based films and reported that zein films had a higher TS and EAB than milk-protein-based films.

Biopolymers can be combined with zein to prepare edible films with desirable properties. A study reported that increasing zein concentration in chitosan-based edible films resulted in increased elasticity of the films with improved barrier properties [48]. Zein films are tough and greaseproof, possessing good barrier properties, but their utilization in food packaging is limited due to poor mechanical characteristics [49].

### 5.3. Wheat Gluten

Wheat gluten is a low-cost protein obtained from the milling industry with good film-forming characteristics, allowing the production of semipermeable membranes capable of reducing water transfer in foods [50]. Wheat gluten-based films could be utilized as food coatings or edible films to delay mass transfer processes such as water and oxygen, which lower food quality [50]. Moreover, the cohesiveness and elasticity of gluten also aid in the film-forming process.

Sharma et al. [51] examined the mechanical attributes of gluten-based films and reported that films with a low amount of glycerol showed increased TS and decreased EAB. However, TS decreased and EAB increased with increasing the glycerol concentration in gluten-based films. Due to the high glutamine content of gluten, which causes the formation of many hydrogen bonds between protein chains, gluten films without the addition of plasticizer are less flexible [51]. Furthermore, the structural, mechanical, and barrier properties of the gluten-based film are influenced by film forming and casting conditions [52].

**Table 2:** Mechanical properties of composite films containing plant proteins.

Plant-Based Protein	Composite Material (CM)	Plasticizer	Tensile Strength (TS)	Elongation at Break (EAB)	References
Zein	Hydroxypropyl Starch (HPS)	Glycerol	TS significantly increased from 14.65 to 17.35 MPa	EAB decreased from 20.42 to 13.33%	[53]
Zein	Tapioca starch	Glycerol	Firmness at Break improved from 9.73 to 39.79 MPa	EAB reduced from 0.64 to 0.30%	[54]
Soy protein Isolate	Galactomannan (GM)	Glycerol	TS increased from 1.88 to 3.72 MPa	EAB decreased from 55.8 to 38.0%	[55]
Soy protein Isolate	Egg white composite (EW) + Cinnamaldehyde (CIN)	Glycerol	TS decreased from 8.05 to 7.23 MPa	EAB increased from 177.42 to 191.70%	[56]
Soy protein Isolate	Edible grasshopper protein + pullulan	Glycerol	TS improved from 3.4 to 7.0 MPa	No positive impact on EAB	[57]

## 6. Mechanical Properties of Animal-Based Protein

The mechanical properties of protein-based materials are intimately related to the nature of the amino acid in the protein [58]. Gelatin, casein, and whey protein are examples of animal-derived proteins having acceptable material qualities. All proteins have complex polymeric structures; however, they are not structurally stable by nature. Using different strategies, the mechanical characteristics of protein-based films can be enhanced, such as by introducing a plasticizer into the protein matrix [59]. Different animal-based proteins including gelatin, casein, and whey protein are utilized in forming edible films due to their significant film-forming characteristics, such as mechanical and barrier characteristics. Table 3 presents the effect of different composite materials on the mechanical parameters of edible films based on animal proteins.

### 6.1. Gelatin

Gelatin is obtained from the partial hydrolysis of natural collagens. Gelatin is an abundant raw material that can be formed at a low cost and has good film-forming characteristics; therefore, it has been utilized in various studies to prepare and examine edible films [60]. The characteristics of the raw materials and the manufacturing processes affect the characteristics of gelatin-based films. Chuaynukul et al. [61] reported that gelatin films made from the casting technique were more resistant to tensile deformation and had a higher level of stiffness when compared to those made via compression molding, regardless of the types of gelatin used.

Mammalian gelatins often exhibit better thermo-stability and physical qualities than most fish gelatins, mainly ascribed to their higher amino acid content [61]. It has also been reported that gelatin films show better transparency, with excellent mechanical and barrier characteristics. Due to its high hygroscopicity, gelatin has a tendency to swell or dissolve when it comes into contact with the surface of meals with a high moisture content. [62]. In a previous study, various concentrations of glycerol and their effect were examined on the mechanical parameter of pigskin gelatin films. The films showed a lower TS and a higher EAB value as the concentration of the glycerol increased [63]. In addition, a number of studies have investigated the effects of incorporating crosslinking and strengthening agents, plasticizers, antimicrobial or antioxidant compounds, and other similar substances into gelatin-based films in order to improve their physiochemical properties.

### 6.2. Casein

Caseins are among the proteins that are excellent for the role of hydrocolloids in the fabrication of edible films because of their excellent nutritional benefits, water solubility, and emulsification ability. Due to the distribution of polar amino acids throughout the protein chain, casein films have unique barrier characteristics that prevent oxygen and other non-polar molecules from penetrating the film [64]. The polar and non-polar amino acid interactions in casein form a rigid film structure during the drying process; therefore, sorbitol or glycerol are added to the films for the plasticizing effects [65]. Casein films are particularly vulnerable to moisture, affecting their mechanical and barrier properties and limiting their applicability as food packaging material [66]. The casein films are not susceptible to denaturation or coagulation, and their stability is sustained over a broad temperature, pH, and salt concentration range. Calcium caseinate films are more rigid and have higher barrier qualities, whilst sodium caseinate films have superior optical properties and EAB but low TS properties [67,68].

Fematt-Flores et al. [69] studied the mechanical parameters of milk-protein-based films, including casein and whey protein isolate (WPI). While all films included 5% milk protein, casein-based films exhibited the lowest TS (0.70 MPa), up to 3.3 times lower than those prepared with WPI. However, casein-based films showed an increased EAB compared to WPI-based films. The mechanical properties of the casein-based edible films can be improved by incorporating different compounds such as crosslinkers and bioactive substances.

### 6.3. Whey Protein

Edible films made from whey protein have been shown to exhibit excellent mechanical and gas barrier properties, even when the relative humidity is relatively low. Whey protein is naturally hydrophilic, which means that these films have a low degree of resistance to moisture [70]. Whey protein is a promising edible biopolymer for food packaging owing to its abundance, safety, biodegradability, and as an environmentally-friendly alternative to synthetic polymers. The two main types of whey protein used in fabricating edible films and coatings are whey protein isolate (WPI) and whey protein concentrate (WPC). Compared to polysaccharides and other protein polymers, films/coatings composed of whey proteins are colorless, odorless, flexible, and transparent, with excellent mechanical and barrier properties [71].

Whey protein films have poor mechanical characteristics because of the high proportion of hydrophilic amino acids present in whey protein [72]. Different treatments, such as adding plasticizing agents, pH modification, and reinforcing nanoparticles, have improved the mechanical properties of whey protein films. With the incorporation of nanocrystalline cellulose (NCC) in the whey protein-based edible films, the TS increased and EAB decreased significantly by increasing the concentration of (NCC) in the film matrix [72]. Jiang et al. [72] reported an increased EAB (86.7%) of WPC-based films when incorporated with transglutaminase (TGase). Furthermore, Fematt-Flores et al. [69] compared the mechanical properties of milk proteins. They reported that whey-protein-based edible films showed a higher TS and a lower EAB than casein-based films.

**Table 3:** Mechanical properties of edible films based on animal proteins.

Animal-Based Protein	Composite Material (CM)	Plasticizer	Tensile Strength (TS)	Elongation at Break (EAB)	References
Gelatin	Casein phosphopeptides (CPPs)	Glycerol	TS increased from 9.60 to 18.14 MPa	EAB increased from 23.4 to 84.1%	[73]
Gelatin	Blood Orange peel pectin (BOPP)	Glycerol	TS increased from 6.23 to 14.36 Mpa	EAB decreased from 10.97 to 4.36%	[74]
Gelatin	Pullulan dialdehyde (PDA)	Glycerol	TS increased from 5.8 to 15.4 MPa	EAB decreased from 471 to 421%	[75]
Whey protein isolate (WPI)	Furcellaran (FUR) + Pu-erh extract (PE) + Greentea extract (GT)	Glycerol	TS increased from 6.87 to 8.20 MPa	EAB decreased from 72.40 to 65.32%	[76]
Whey protein isolate (WPI)	γ-Aminobutyric acid (GABA)	Glycerol	TS decreased with the addition of GABA	EAB increased with the addition of GABA	[77]

## 7. Conclusions

Edible films and coatings made of protein have great promise for enhancing food quality, shelf life, functionality, and safety. The intrinsic qualities of the proteins, plasticizers, and other chemicals added for various purposes, such as cross-linkers, antimicrobials, antioxidants, etc., as well as the film formation technique and treatment conditions, significantly impact the effectiveness and properties of protein films. Moreover, the mechanical properties of edible films are crucial for their performance as packaging materials, as they determine their strength, flexibility, and ability to protect the food product. Furthermore, studies are needed to examine and enhance the mechanical properties of protein-based food packaging materials for industrial applications.

## Figures and Tables

**Table 1:** Instruments to assess the mechanical strength of the food packaging material.

Model	Manufacturer	Sample Nature		Purpose	Reference
Attopuls Texture Analyser	Stable Micro Systems, UK	Starch-based (potato, corn, sweet potato, green bean and tapioca) edible packaging film incorporated with blueberry pomace powder	tensile grip (TA 96B) attachment, initial grip separation of 20 mm, and a pre-test and test speed of 1 mm/s.	To determine the tensile strength	[19]
Zwick Roell Texture machine mod. Z2.5	ZwickRoell, Ulm, Germany	Edible Films based on Citral Essential Oil, Alginate and Pectin	500 N load cell, pre-load of 1MPa with a pre-load speed of 5 mm/min, crosshead speed of 5 mm/min.	Elastic modulus, stress at yield and at break, and elongation at yield and at break	[20]
CT3, Brookfield Engineering Laboratories Inc.	Middleboro, MA, USA	Edible Films Based on Whey Protein Isolate and Tarragon Essential Oil	puncture head (a cylindrical rod of 2 mm in diameter-TA39), was set to a target distance of 5.0 mm with a speed of 0.5 mm/s.	puncture resistance and puncture deformation	[21]
QMESYS Universal Material Testing Machine, QM100s, 1.96 kN	Komachine, Gyeonggi-do, Korea	Mucilage polysaccharides (OLP) and carboxymethyl cellulose (CMC) extracted from okra leafstalk wastes	gauge distance and crosshead speed were set at 20 mm and 10 mm/s	tensile strength (TS) and elongation at break (EB)	[22]
Nanoindenter	CSM, Peseux, Switzerland	Chitosan–Zein Edible Films with Added Essential Oils	load of 2.5 mN at loading and unloading rates of 7.5 mN/min, and a pause of 35 s using a Berkovich tip	Elastic modulus	[23]
Auto tensile tester	XLW (EC) or XLW-PC, China	tef starch based edible films		tensile strength, elongation at break, elastic modulus, puncture force, and puncture deformation	[24]
AG-IS 50kN-Universal texture Machine	Shimadzu AG-IS 50kN, Kyoto, Japan	Edible Film from Fermented Cheese Whey and Cassava Peel Starch		elongation at break and tensile strength	[25]
Type HT-8503, Universal Testing Machine	Seremban, Negeri Sembilan, Malaysia	Bio-nano composite gelatin-based edible film by combining nanogelatin, cellulose nanocrystal and nanopropolis fillers		tensile strength, elongation at break, and Young’s modulus	[26]
INSTRON 3345 universal testing machine	High Wycombe, UK	Opuntia ficus-indica mucilage	a head speed of 100 mm/min using a double clamp with a separation of 50 mm	tensile strength, elastic modulus and elongation at break	[27]

## Data Availability

Not applicable.
